# Change in CD3 positive T-cell expression in psoriatic arthritis synovium correlates with change in DAS28 and magnetic resonance imaging synovitis scores following initiation of biologic therapy - a single centre, open-label study

**DOI:** 10.1186/ar3228

**Published:** 2011-01-27

**Authors:** Eliza K Pontifex, Danielle M Gerlag, Martina Gogarty, Marjolein Vinkenoog, Adrian Gibbs, Ilse Burgman, Ursula Fearon, Barry Bresnihan, Paul Peter Tak, Robin G Gibney, Douglas J Veale, Oliver FitzGerald

**Affiliations:** 1 Department of Rheumatology, St. Vincents University Hospital, Elm Park, Dublin 4, Ireland; 2 Division of Clinical Immunology and Rheumatology F4-218, Academic Medical Center/University of Amsterdam, PO Box 22700, 1100 DE Amsterdam, The Netherlands; 3 Department of Radiology, St. Vincents University Hospital, Elm Park, Dublin 4, Ireland

## Abstract

**Introduction:**

With the development of increasing numbers of potential therapeutic agents in inflammatory disease comes the need for effective biomarkers to help screen for drug efficacy and optimal dosing regimens early in the clinical trial process. This need has been recognized by the Outcome Measures in Rheumatology Clinical Trials (OMERACT) group, which has established guidelines for biomarker validation. To seek a candidate synovial biomarker of treatment response in psoriatic arthritis (PsA), we determined whether changes in immunohistochemical markers of synovial inflammation correlate with changes in disease activity scores assessing 28 joints (ΔDAS28) or magnetic resonance imaging synovitis scores (ΔMRI) in patients with PsA treated with a biologic agent.

**Methods:**

Twenty-five consecutive patients with PsA underwent arthroscopic synovial biopsies and MRI scans of an inflamed knee joint at baseline and 12 weeks after starting treatment with either anakinra (first 10 patients) or etanercept (subsequent 15 patients) in two sequential studies of identical design. DAS28 scores were measured at both time points. Immunohistochemical staining for CD3, CD68 and Factor VIII (FVIII) was performed on synovial samples and scored by digital image analysis (DIA). MRI scans performed at baseline and at 12 weeks were scored for synovitis semi-quantitatively. The ΔDAS28 of the European League Against Rheumatism good response definition (>1.2) was chosen to divide patients into responder and non-responder groups. Differences between groups (Mann Whitney U test) and correlations between ΔDAS28 with change in immunohistochemical and MRI synovitis scores (Spearman's rho test) were calculated.

**Results:**

Paired synovial samples and MRI scans were available for 21 patients (8 anakinra, 13 etanercept) and 23 patients (8 anakinra, 15 etanercept) respectively. Change in CD3 (ΔCD3) and CD68 expression in the synovial sublining layer (ΔCD68sl) was significantly greater in the disease responders compared to non-responders following treatment (*P *= 0.005 and 0.013 respectively). ΔCD3, but not ΔCD68 or ΔFVIII, correlated with both ΔDAS28 (r = 0.49, *P *= 0.025) and ΔMRI (r = 0.58, *P *= 0.009).

**Conclusions:**

The correlation of ΔCD3 with ΔDAS28 and ΔMRI following biologic treatment in this cohort contributes to the validation of ΔCD3 as a synovial biomarker of disease response in PsA, and supports the further evaluation of ΔCD3 for predictive properties of future clinical outcomes.

## Introduction

Psoriatic arthritis is a chronic and debilitating inflammatory arthropathy. It accounts for 15% of referrals to early arthritis clinics, and has considerable morbidity [1]. The Outcome Measures in Rheumatology Clinical Trials (OMERACT) PsA working group has identified a hierarchy of domains to be included in PsA clinical trials [2], which includes tissue analysis and magnetic resonance imaging (MRI) in the outer domain, on the research agenda. Utilizing these two domains, we have sought a potential synovial biomarker of treatment response in PsA. A biomarker is defined as a characteristic that is objectively measured and evaluated as an indicator of a normal biologic process, a pathophysiologic process, or a pharmacological response to therapeutic intervention [3].

It has already been established in rheumatoid arthritis (RA) that the mean change in DAS28 correlates with the mean change in synovial sublining CD68 expression across several RA patient cohorts receiving different therapeutic agents [4-7]. Few studies have correlated clinical composite scores with changes in PsA synovial cell populations however. One of the reasons for this is that no composite score has yet been validated in PsA, although such work is currently in progress [8]. DAS28, a score validated in RA [9], has proven to be a highly effective tool in previous studies of PsA and biologic agents [10-12] and is suitable for studies involving synovial tissue analysis as it focuses on articular involvement. In the synovial tissue of our patient cohort, we measured the expression of CD68, a macrophage marker, given the clinical correlations found in RA; FVIII, an endothelial cell marker, due to the hypervascularity and vessel tortuosity evident in inflammed PsA synovium compared to that of RA [13-16]; and CD3, a T-cell marker. Importantly, a previously published study which utilized DAS28 found a significant correlation between ΔDAS28 and ΔCD3 in the synovium of patients with PsA after adalimumab treatment [12]. Should this finding prove reproducible, particularly if different therapeutic agents are used, ΔCD3 may meet the discrimination criterion of the OMERACT biomarker validation filter [17] and the exploration of ΔCD3 as a predictive biomarker of future treatment response in PsA would be supported. ΔCD3 could be used to determine the potential efficacy of new therapeutic agents in PsA at an early stage, as is already happening in RA clinical trials of novel therapeutic compounds, where synovial sublining ΔCD68 measurements are being observed to reflect clinical response [18,19].

While MRI has been used to highlight the importance of bone marrow oedema and entheseal sites of inflammation in PsA [20,21], to date there have been no studies comparing histological change with quantified synovitis by dynamic or static MRI. In this study we examine the relationship between clinical scores and both immunohistochemical (IHC) and MRI measures of synovitis following biologic treatment in PsA to help identify a potential biomarker of treatment response.

## Materials and methods

### Study protocol

Twenty-five patients who met the CASPAR classification criteria for PsA [22] were enrolled in two sequential studies of identical design. The first 10 consecutive patients received anakinra, an IL-1 receptor antagonist, 100 mg by subcutaneous injection (SC) daily, followed by 15 consecutive patients who received etanercept, a TNF receptor antagonist, 25 mg twice weekly SC. Both were 12-week, single centre, open-label studies undertaken at St. Vincents University Hospital, Dublin. Ethical approval was obtained by the hospital's ethics committee and written informed consent was provided by all patients. At the time of enrolment, each patient had to have a diagnosis of PsA for at least three months, and at least three tender and three swollen joints (one of which was a knee), of a 68-joint assessment,.

Clinical parameters were measured at weeks 0 and 12, including 28 and 68 tender (TJC) and 28 and 66 swollen joint counts (SJC), patient pain and disease 0 to 100 mm visual analogue scale (VAS) and the Health Assessment Questionnaire (HAQ). Serum erythrocyte sedimentation rate (ESR) (Test-1, Ali Sax) and C-reactive protein (CRP) levels (nephelometry) were also measured. A DAS28 score was calculated at each time point. To look for changes in cell marker expression and MRI synovitis scores reflecting change in clinical activity, the change in DAS28 of the EULAR definition of good response (>1.2) [23] was chosen to divide the cohort into two groups, labelled here as responders (ΔDAS28 >1.2) and non-responders (ΔDAS28 ≤1.2).

To compare the single joint MRI synovitis scores with a single joint clinical measure, a more detailed clinical assessment was performed of each patient's involved knee. It was scored in the manner of the first published study of PsA and a biologic agent in which pain and swelling were evaluated separately on a scale of 0 to 3, where 0 represents the absence of pain or swelling [24]. The sum of these is the final score for a given joint, which will range from 0 to 6. A patient was defined as a knee responder in this study if there was a reduction in their involved knee score following treatment at Week 12.

Patients were excluded if they had received a biologic agent within 12 weeks, cyclosporin or leflunomide within 8 weeks, or methotrexate or sulfasalazine within 4 weeks of enrolment into the study. Patients taking ≥10 mg of prednisolone or those who had a prednisolone dose change within four weeks of study Day 1 were also excluded, as were those who were pregnant, breastfeeding, had significant liver, renal or haematological abnormalities, or a history of cancer within five years of the study's commencement. Prior to receiving etanercept, patients were screened for latent tuberculosis with a chest X-ray and Mantoux test.

### Arthroscopy

Arthroscopy and synovial biopsy of the involved knee joint was performed at two time points (weeks 0 and 12), with a Storz arthroscope and 1.5 mm grasping forceps. Biopsy samples were obtained from all knee joint compartments, embedded in TissueTek OCT compound (Sakura, Alphen aan den Rijn, Netherlands) and stored in liquid nitrogen. In the majority of cases, six individual biopsies were included together in one OCT mould for cutting and analysis. Seven μm thick sections were cut using a cryostat, placed on glass slides coated with 2% 3-amino-propyl-triethoxy-silane (Sigma-Aldrich Ireland Ltd, Dublin, Ireland) and dried overnight at room temperature. Sections were stored at -80°C until required for staining.

### Immunohistochemistry

A routine three-stage immunoperoxidase labeling technique was used. Tissue sections were allowed to reach room temperature, were fixed in acetone for 10 minutes and then air-dried. The remaining steps were performed in an autostainer using reagents from an Envision+ system-HRP (AEC) kit (Dako, Glostrup, Denmark). Following quenching of endogenous peroxidase activity, the synovial sections were incubated with mouse monoclonal antibodies against cell specific markers CD3, CD68, and FVIII (Dako, Glostrup, Denmark) for 30 minutes. Incubation with a labelled polymer-HRP anti-mouse antibody followed, and colour was then developed with amino-ethylcarbazole (AEC). Slides were counterstained with Mayer's haematoxylin (BDH Laboratories, Poole, UK) and mounted.

### Digital image analysis

All slides were randomly assigned code numbers for analysis and only tissue samples with a clearly identifiable intimal lining layer were included. All analysis was undertaken by EKP. Eighteen high power images were taken per slide for each of the three cell specific markers stained. In the case of CD68, the intimal lining layer was highlighted manually per image, such that staining could be quantified in two areas - the intimal lining (ll) and synovial sublining (sl) layers. Analysis was performed using the Qwin analysis system (Leica, Cambridge, UK) as has been previously described [25,26]. Results are expressed as the number of positively stained cells/mm^2 ^of tissue for CD3 and CD68, and by integrated optical density (IOD)/mm^2 ^for FVIII. The average value over all six biopsies per patient per time point was used for analysis.

### MRI

An MRI scan of the same involved knee was performed for each patient the day prior to arthroscopy at weeks 0 and 12, using a 1.5T Signa Excite HD MRI scanner (General Electric Healthcare, Chalfont St Giles, Buckinghamshire, UK) and a dedicated eight channel array HD knee surface coil with patients lying supine. The examinations performed included intravenous contrast enhanced (Gadoteric acid, Dotarem, 0.5 mmol/mL, Guerbet Laboratories, Birmingham, UK); 10 mls in all examinations by slow hand injection) T1-weighted fat suppressed pulse sequences in coronal, sagittal and axial planes. Scanning parameters were as follows: coronal, TR 640 ms; TE 16; slice thickness 4/1 mm; FOV 18; NEX 2; matrix 512 × 256; sagittal, TR 500; TE 16; slice thickness 4/1 mm; FOV 22; NEX 2; matrix 256 × 192 axial, TR 440; TE 11; slice thickness 3/1.5 mm; FOV 16; NEX 3; matrix 224 × 192.

Once complete, the scans were arranged into pairs of pre- and post-treatment images for each patient. These were scored semi-quantitatively by one consultant radiologist with a special interest in musculoskeletal radiology who was blind to patient identity and scan chronology. Each knee was divided into four anatomical regions (medial and lateral parapateller recesses, intercondylar notch and suprapatellar pouch) and a synovitis score ranging from 0 to 3 was assigned to each region (0 = normal synovium, 1 = diffuse, even thickening, 2 = nodular thickening, 3 = gross, nodular thickening) based on the overall impression of the severity of synovial abnormality in the three orthogonal scanning planes. This method has been described and validated for synovitis in knee osteoarthritis by Rhodes *et al. *[27]. The regional scores were added for a final synovitis score per knee ranging from 0 to 12.

### Statistical analysis

Data was analysed with SPSS 12.0.1 for Windows (SPSS Inc, IBM, Chicago, Illinois, USA). Change in clinical parameters, IHC markers and MRI synovitis scores following treatment were evaluated using the Wilcoxon signed rank test and differences between responder and non-responder groups were determined with the Mann Whitney U test. Correlations between ΔDAS28 with change in IHC and MRI synovitis scores were calculated using Spearman's rho test.

## Results

Twenty-five patients completed at least one of the IHC or MRI components of these studies (19 completed all components), and were, therefore, included for clinical analysis (10 anakinra, 15 etanercept). Patients were 50% and 66.6% female, had a mean age (range) of 43.2 (27 to 60) and 48.7 (26 to 64) years and a mean disease duration of 9.1 (1 to 42) and 7.5 (1 to 29) years in the anakinra and etanercept treated cohorts, respectively. Four patients had oligoarthritis (≤4 involved joints) at enrolment (all etanercept treated); the remaining 21 patients had polyarticular disease (mean SJC66 17, SD 9.2, range 6 to 43).

Five of the anakinra patients (50%) and 11 of the etanercept patients (73.3%) were taking a non-steroidal anti-inflammatory drug (NSAID), 2 and 4 of whom were also on a stable dose of prednisolone ≤10 mg.

### Clinical responses

Changes in clinical parameters following treatment are shown in Table 1. In both studies, the DAS28 was reduced significantly after treatment; the changes were more pronounced in the etanercept group compared to the anakinra group. Nineteen of the 25 patients achieved an improvement in DAS28 of >1.2 and are labelled responders (5 anakinra and 14 etanercept), and 6 patients did not and are labelled non-responders (5 anakinra, 1 etanercept).

**Table 1:** Clinical parameters of patients with PsA at baseline and 12 weeks following treatment

	Anakinra n = 10			Etanercept n = 15		
	Week 0	Week 12	*P-*value	Week 0	Week 12	*P-*value
TJC28	9.5 (3 to 19)	7 (1 to 13)	0.033	11 (1 to 25)	2 (0 to 28)	0.004
TJC68	24.5 (9 to 52)	13.5 (3 to 35)	0.015	15 (3 to 57)	3 (0 to 38)	0.002
SJC28	8 (1 to 18)	5 (0 to 8)	0.032	4 (1 to 21)	1 (0 to 8)	0.005
SJC68	20.5 (6 to 27)	8 (0 to 23)	0.028	9 (3 to 43)	2 (0 to 16)	0.007
ESR	18.5 (3 to 79)	7.5 (2 to 46)	0.059	14 (4 to 91)	5 (1 to 26)	0.001
CRP	17 (4 to 70)	7.5 (0 to 29)	0.044	7 (0 to 42)	0 (0 to 18)	0.092
d VAS	66 (27 to 85)	47 (12 to 70)	0.051	44.5 (5 to 93)	15 (2 to 56)	0.002
p VAS	65.5 (21 to 91)	46.5 (20 to 75)	0.169	50 (22 to 93)	12 (2 to 66)	0.001
HAQ	1.25 (0.75 to 2.38)	1.13 (0.25 to 1.88)	0.057	1.13 (0 to 2.5)	0.25 (0 to 2.25)	0.01
DAS28	5.03 (3.77 to 7.16)	4.17 (2.35 to 5.98)	0.022	5.26 (3.08 to 6.95)	2.01 (0.14 to 5.35)	0.001

Median (range). d VAS, disease visual analogue scale; p VAS, pain visual analogue scale.

Twenty-three patients had involved knee scores available. There was a significant difference between the knee scores of the knee responders (n = 16, 8 anakinra, 8 etanercept) at Week 0 and Week 12 (3 (1 to 6) and 1 (0 to 4) respectively, median (range), *P *= 0.00), and not between the non-responders (n = 7, 2 anakinra, 5 etanercept), (2 (2 to 3) and 3 (2 to 4) respectively, *P *= 0.1).

### Immunohistochemistry

Synovial biopsies at baseline and 12 weeks were available for 21 patients (8 anakinra, 13 etanercept).

**Figure 1:**
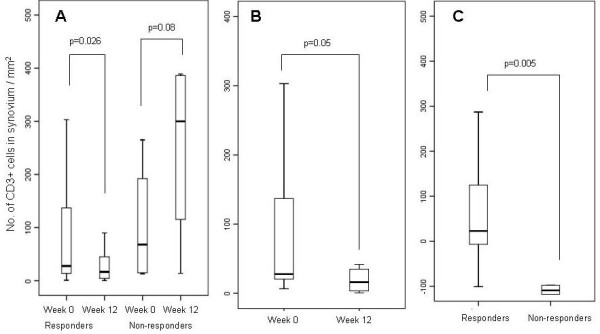
**ΔCD3 of combined responders and non-responders (A), etanercept responders (B). and ΔCD3 of combined responders versus non-responders (C)**.

Combining the total patient cohort, there was a significant reduction in CD3 expression following treatment in the responder group (28 (1 to 1,344) at Week 0 to 17.5 (0.5 to 734) at Week 12, *P *= 0.026, median (range)), but not in the non-responder group (68 (13 to 265) at Week 0 and 217 (14 to 389) at Week 12, *P *= 0.080) (Figure 1A). A reduction in expression was not observed for any of the other cell markers following treatment in either the responder or non-responder groups. Of interest, however, there was a significant increase in CD68sl expression in the non-responder group at Week 12 (1,835 (1 667 to 2,218)) compared to Week 0 (1,409 (494 to 1,795)), (*P *= 0.043).

The degree of change in cell marker expression following treatment was significantly greater for ΔCD3 in the group of responders (19 (-100 to 1,031)) than the non-responders (-109.3 (-376 to 3)), *P *= 0.005, (Figure 1C). This was also the case for ΔCD68sl (-53 (-1,336 to 2,178)) among the responders compared to the non-responders (-382 (-1,247 to -127)), *P *= 0.013.

Looking at the individual treatment groups separately, there was a significant reduction in CD3 expression in the etanercept treated responders at Week 12 (n = 16), but not CD68sl, CD68ll or FVIII (Figure 1B and Table 2). In the anakinra treated patients, there was no change within the responder group in CD3 expression, but there was a non-significant increase in the non-responder group following treatment.

**Table 2:** Change in cell marker expression following treatment in the responder and non-responder groups

			Week 0	Week 12	*P-*value
CD3	etanercept	R n = 12	27.5 (6.5 to 1121)	16 (0.5 to 113)	0.05*
		NR n = 1	17	14	n/a
	anakinra	R n = 4	33.5 (1 to 1344)	32.9 (3 to 734)	0.47
		NR n = 4	87.9 (13 to 265)	300 (166 to 389)	0.068
CD68sl	etanercept	R n = 12	127 (138 to 3,543)	712 (112 to 2,318)	0.31
		NR n = 1	1,408	1,667	n/a
	anakinra	R n = 4	1,370 (362 to 2,685)	1,444 (453 to 2,414)	0.72
		NR n = 4	1,431 (494 to 1,795)	1,879 (1,741 to 2,218)	0.068
CD68ll	etanercept	R n = 12	226 (38 to 513)	213 (17 to 466)	0.48
		NR n = 1	191	150	n/a
	anakinra	R n = 4	174 (129 to 469)	214 (154 to 473)	0.47
		NR n = 4	159 (108 to 197)	147 (104 to 319)	0.72
FVlll	etanercept	R n = 11	132,242 (43,272 to 754,550)	139,294 (51,817 to 439,712)	0.53
		NR n = 1	58,525	142,834	n/a
	anakinra	R n = 4	218,619 (88,372 to 353,725)	280,785 (226,415 to 353,725)	0.27
		NR n = 4	286,939 (84,419 to 521,103)	437,447 (184,155 to 545,675)	0.72
MRI	etanercept	R n = 13	8 (4 to 12)	5 (2 to 11)	0.12
		NR n = 1	3	1	n/a
	anakinra	R n = 5	4 (4 to 12)	4 (4 to 9)	0.66
		NR n = 3	4 (0 to 11)	4 (4 to 7)	1

Median (range). n/a, not applicable; NR, non-responder; R, responder

### MRI

Paired baseline and 12-week scans were available for 23 patients (8 anakinra, 15 etanercept).

There was no change in MRI detected synovitis following treatment in the combined cohort of responders (5 (4 to 12) at Week 0 to 5 (2 to 11) at Week 12, *P *= 0.1) or non-responders (3.5 (0 to 11) to 4 (1 to 7), *P *= 0.79). Likewise, there was no difference in the change in MRI synovitis scores following treatment between the responder and non-responder groups (0 (-3 to 6) and 1 (-7 to 7) respectively, *P *= 0.83). Individually, neither etanercept nor anakinra treatment led to a significant difference in MRI synovitis scores in either the responder or non-responder groups (Table 2).

Looking specifically at the knee responders, there was a significant difference in the MRI synovitis scores of the knee responders (n = 15) at Week 0 compared to Week 12 (6 (4 to 12) and 4 (2 to 11), *P *= 0.049), but not of the knee non-responders (n = 5), (4 (0 to 8) and 4 (1 to 7), *P *= 1.0).

### Associations between ΔCD3, ΔDAS28 and ΔMRI

The primary aim of this study was not to compare the clinical efficacy or specific effects on the synovium of two different biologic agents, but to seek a candidate biomarker of disease response. Change in this biomarker should correlate with change in disease activity and be irrespective of the type of therapeutic intervention used. All patient data were combined, therefore, to determine correlations between change in DAS28 with change in IHC and MRI synovitis scores, as has been done in previous similar studies [12,28].

**Figure 2:**
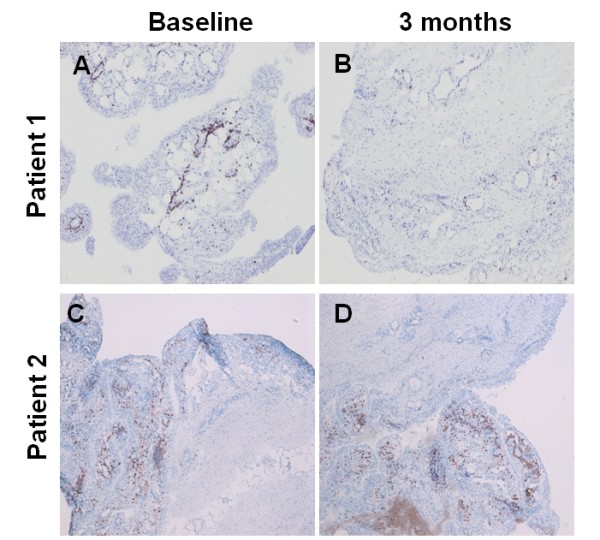
**Synovial images showing CD3 expression in a DAS28 moderate responder (Patient 1) and non-responder (Patient 2)**. **A **and **B **are baseline and Week 12 images of Patient 1, and **C **and **D **are baseline and Week 12 images of Patient 2 respectively.

ΔCD3 expression correlated significantly with ΔDAS28 following treatment (r = 0.49, *P *= 0.025), (Table 3). No correlations were observed between ΔDAS28 or any of its individual components, and change in expression of the other IHC markers. Figure 2 shows representative images of synovial CD3 expression at baseline and 12 weeks for two patients with differing clinical responses. Patient 1 (etanercept) achieved a ΔDAS28 of 1.22 and ΔCD3 of 19, and Patient 2 (anakinra) achieved a ΔDAS28 of 0.16 and ΔCD3 of -118.

**Table 3:** Correlation of ΔDAS28 and ΔMRI synovitis scores with cell marker expression following biologic treatment

	ΔCD3	ΔCD68 sl	ΔCD68 ll	ΔFVIII
ΔDAS28	0.49 (0.025*)	0.27 (0.24)	-0.07 (0.77)	0.244 (0.30)
ΔMRI	0.58 (0.009*)	0.22 (0.378)	0.07 (0.78)	0.33 (0.18)

Correlation coefficient (*P *=). N = 21 for all IHC groups except FVIII (n = 20) and n = 19 for all MRI groups; ll, lining layer; sl, sublining layer.

**Figure 3:**
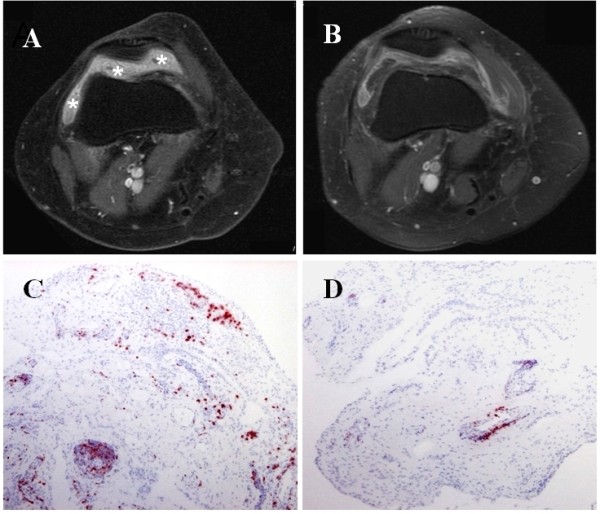
**Baseline (A) and Week 12 (B) MRI scans with corresponding baseline (C) and Week 12 (D) CD3 stained synovium (red-brown)**. Thickened enhanced synovium (* in A) has improved following treatment.

There was a significant correlation between ΔCD3 and ΔMRI synovitis following treatment (r = 0.58, *P *= 0.009), (Table 3). Furthermore, MRI synovitis and CD3 expression measured at all time points correlated significantly (r = 0.504, *P *= 0.001), (n = 38). ΔMRI did not correlate with change in expression of the other IHC markers or with ΔDAS28 scores (r = -0.027, *P *= 0.91). Figure 3 shows representative images of CD3 stained synovium and corresponding MRI scans of a patient who had a ΔDAS28 of 2.54, a ΔCD3 score of 287 and a ΔMRI synovitis score of 4.

## Discussion

This study has demonstrated that change in synovial CD3+ T-cell expression correlates with both ΔDAS28 and ΔMRI synovitis scores in a cohort of patients with PsA treated with either anakinra or etanercept.

Over the last 15 years, some fundamental features of the spondyloarthropathy (SpA) synovium have been elucidated. First, the inflamed synovium of SpA appears to differ histologically from that of RA [13-16,29]. Second, the synovial histology of subtypes of SpA, including oligoarticular versus polyarticular PsA, have been shown to be similar [16,29,30]. Third, there are histological changes in the synovium when patients with SpA respond to effective treatment. Two studies of anti-TNF therapy in SpA have shown a significant reduction in polymorphonuclear (PMN) cells, CD4+ and CD8+ T cells and macrophage subsets after 12 weeks, plus a trend toward reduced CD3+ T cell numbers [31,32]. Exclusively in PsA, reduction in T cell and sublining macrophage infiltration has been observed as early as 48 hours after an infliximab infusion [33] and also following treatment with alefacept and methotrexate [34,35]. Correlations with clinical outcomes were not performed in these studies. Consistent with our etanercept responders, in a cohort of PsA patients treated with adalimumab, a significant reduction in the number of CD3 positive cells was observed after four weeks [12]. The number of CD68+ cells in the synovial sublining did decrease in the responders of both latter studies, but not significantly, while CD68sl expression in this current study's non-responders significantly increased. Thus, in PsA, change in synovial CD3 cell infiltration, and not CD68, appears to be a superior biomarker of treatment response. Reduction in angiogenesis has been demonstrated in PsA patients after infliximab treatment [36,37], but was not found after etanercept treatment in the present study, and may be related to the difference in mechanism of action between the anti-TNF antibodies compared to etanercept [38].

Only two previously published trials of SpA synovium have measured DAS28 scores. Of 52 SpA patients who may have received infliximab, etanercept or no biologic treatment, DAS28 scores were calculated for 28 patients who had polyarticular disease [28]. These scores correlated only with change in CD163 expression, a macrophage subset marker, in the lining layer, and not with change in CD3 or CD68 expression in the sublining layer. The patients with PsA were not evaluated as a distinct group. Only one other trial, which used adalimumab or placebo, has exclusively enrolled PsA patients and used DAS28 as a primary clinical outcome measure [12]. Consistent with our results, that study also demonstrated a significant correlation between ΔCD3 and ΔDAS28. Taking these two papers together, four different agents have now been used in two PsA cohorts (anakinra/etanercept and adalimumab/placebo) and both studies have found this proportional relationship between ΔCD3 and ΔDAS28.

While not conclusive, the findings in this study are also consistent with the hypothesis that T-cells play an active role in PsA pathogenesis. Large numbers of T cells are present in the PsA synovium, synovial fluid and subchondral bone beneath the entheses [13,29,39], where bone oedema and erosions can occur. Th1 derived cytokines dominate in the PsA synovium [35,40] and the CD8+ cell population contains T cell repertoires which are oligoclonally expanded [41]. The association of PsA with human leukocyte antigen (HLA) Class 1 [42], the development of Ps and PsA as a manifestation of the Acquired Immunodeficiency Syndrome (AIDS) and the transmission of PsA following bone marrow transplantation [43,44] all suggest T-cells take part in disease expression. Lastly, the fact that cyclosporin and ustekinumab, which impair T-cell activation, and alefacept, which specifically targets activated T cells, are effective in PsA, support T-cell involvement further [45-47].

The use of MRI in PsA research has been reviewed in detail [48], and an MRI scoring system for hands in PsA has recently been developed by the OMERACT imaging group [49,50]. We opted for our scoring method as it focuses on knee synovitis and is semi-quantitative. MRI synovitis has been shown to reduce significantly following anti-TNF therapy in PsA [51,52], and we found this to be the case in our combined group of clinical knee responders. These former studies assessed mostly hand joints as opposed to exclusively knees and used quantitative analysis. Correlations of MRI findings and histopathology in inflammatory arthritis are emerging in the literature. Bollow *et al. *compared dynamic MRI and sacroiliac joint immunohistochemistry [53] and found T cells and macrophages to be the most common inflammatory cells in active SpA sacroiliitis, although >95% of the tissue obtained was cartilage and bone. In severe AS, MRI-detected bone oedema has correlated well with histological bone marrow oedema of zygoapophyseal joints, but less so with actual inflammatory cell infiltrates [54]. Other studies correlating MRI findings with synovial and bone oedema histology have been undertaken in RA [55,56], but not yet in PsA. This is the first study, therefore, to demonstrate a relationship between MRI synovitis and CD3 expression in PsA, both at all time points in the study and when comparing the changes with treatment.

No relationship was found in this cohort between ΔMRI synovitis and ΔDAS28. The fact that this MRI data reflects change in a single joint only, in contrast to DAS28, and that improvement in DAS28 may or may not involve the knee, will contribute to this. The strongest correlation being between ΔCD3 expression and ΔMRI synovitis scores is not surprising, as these originate from the same single joint and are objectively measured, excluding any subjectivity of clinical scoring and additional pathologies that could influence single joint clinical scores.

As two patients did not undergo follow-up MRI and three different patients did not have adequate synovial tissue for analysis, our study is limited by some uncoupling of the patient groups included in IHC and MRI analyses. Also, in three pre-treatment scans (two etanercept, one anakinra), insufficient fat suppression may have led to an underestimation of the degree of synovitis.

## Conclusions

This study demonstrates a significant correlation between synovial ΔCD3 expression and ΔDAS28, and synovial ΔCD3 expression and ΔMRI synovitis scores in a cohort of 25 patients with PsA treated with either anakinra or etanercept. The establishment of ΔCD3 as a candidate biomarker of treatment response in PsA should prompt other studies using different therapeutic agents to reinforce this concept, and also to determine its ability to predict future clinical outcomes. Further work focusing on changes in peripheral blood T-cell subsets for a more easily accessible biomarker could prove useful.

## Abbreviations

Δ: change in; AEC: amino-ethylcarbazole; CASPAR: Classification of psoriatic arthritis; CRP: C-reactive protein; d VAS: disease visual analogue scale; DAS28: disease activity score assessing 28 joints; DIA: digital image analysis; EULAR: European League Against Rheumatism; FVIII: Factor VIII; HAQ: health assessment questionnaire; IHC: immunohistochemistry; IL-1: interleukin 1; IOD: integrated optical density; ll: synovial lining layer; MRI: magnetic resonance imaging; n/a: not applicable; NR: non-responder; OMERACT: outcome measures in rheumatology clinical trials; pVAS: pain visual analogue scale; PsA: psoriatic arthritis; R: responder; RA: rheumatoid arthritis; SJC: swollen joint count; sl: synovial sublining layer; SpA: spondyloarthropathy; TJC: tender joint count; TNF: tumour necrosis factor; VAS: visual analogue scale.

## Competing interests

UF had grant research support from GlaxoSmithKline and is a consultant for and received grant research support from Wyeth. PPT is a consultant for Abbott, Amgen, Schering-Plough, and Wyeth. DJV received grant research support from GlaxoSmithKline, is a consultant for and received grant research support from Schering-Plough, is a site primary investigator for Roche, and is a consultant for and received grant research support from Wyeth. OF received grant research support from Abbott, is a primary investigator for Bristol Myers Squibb, and received grant research support from Wyeth.

The other authors declare that they have no competing interests.

## Authors' contributions

EKP cut and stored the etanercept slides and performed the initial IHC staining, performed the digital image analysis, collated all data, performed all statistical analysis and wrote the manuscript. DMG participated in the autostainer immunohistochemistry. MG cut and stored all anakinra slides and performed initial IHC staining. MV made a substantial contribution to the DIA part of this work and arranged DIA data ready for analysis. AG was involved in patient recruitment and clinical assessment. IB participated in the autostainer immunohistochemistry. UF made a substantial contribution to the statistical analysis. BB made a substantial contribution to the conception and design of the study. PPT has been involved in the DIA aspect of this work and revising the manuscript critically for content. RG arranged the MRI scanning and performed the MRI synovitis scoring. DV made a substantial contribution to the conception and design of the study and coordinated the arthroscopies. OF conceived of the study, participated in its design and coordination and has been involved in the revision of the manuscript critically for content. All authors have read and approved the final version of this manuscript.
